# Allometric relationships between leaf and bulb traits of *Fritillaria przewalskii* Maxim. grown at different altitudes

**DOI:** 10.1371/journal.pone.0239427

**Published:** 2020-10-05

**Authors:** Ruili Ma, Shengrong Xu, Yuan Chen, Fengxia Guo, Rui Wu

**Affiliations:** 1 Qinghai University Medical College, Qinghai Provincial Key Laboratory of Traditional Chinese Medicine Research for Glucolipid Metabolic Diseases, Xi′ning, China; 2 College of Agronomy, Gansu Provincial Key Laboratory of Aridland Crop Science, Gansu Key Laboratory of Crop Genetic & Germplasm Enhancement, Gansu Provincial Key Lab of Good Agricultural Production for Traditional Chinese Medicines, Gansu Provincial Engineering Research Centre for Medical Plant Cultivation and Breeding, College of Life Science and Technology, Gansu Agricultural University, Lanzhou, China; Beijing Forestry University, CHINA

## Abstract

Plants adapt to high altitudes by adjusting the characteristics of their above and underground organs. Identifying the species-specific plant traits changed in response to altitude is essential for understanding ecophysiological processes at the ecosystem level. Multiple studies analyzed the effects of altitude on above and underground organ traits in different species. Yet, little is known about those responses in the alpine *Fritillaria przewalskii* Maxim. (Liliaceae). *F*. *przewalskii* is a perennial medicinal plant with meager annual growth and vanishing wild populations. We analyzed leaf and bulb functional traits, and their allometric relationships in *F*. *przewalskii* plants grown at three altitudes: 3000, 2700, and 2400 m. Leaf thickness, leaf biomass, leaf biomass allocation, and the aboveground:underground ratio increased significantly with increasing altitude. Conversely, bulb allocation decreased at higher altitudes. The altitude influenced the allometric growth trajectories of specific leaf and bulb traits: higher altitudes led to thicker and broader leaves and changed the shape of the bulbs from more circular, which is ideal (at 2700 m), to more elongated (at 3000 m). Those variations had remarkable ecological significance. Hence, bulb biomass is the largest at 2700 m of altitude for which their vertical and longitudinal ratio is unaffected. which is economically favorable. Our findings show that *F*. *przewalskii* has a notable potential of growth and morphological plasticity along the altitude gradient and that 2700 m might be ideal for developing its artificial cultivation.

## Introduction

Total plant growth results from an unequal increase in biomass among different organs [1]. How plants allocate resources depends on the species and results from long-term adaptation to their ecological niches. While organ-specific biomass allocation is mainly controlled by genetic traits, it is also modulated by the changing environment [2]. Hence, it reflects the ability of plants, as sessile organisms, to use the resources available and to adapt to heterogeneous conditions [3–5]. The distribution patterns of biomass directly affect plant growth and reproduction strategies [6].

Huxley first introduced the concept of allometry to the life sciences after observing that the relative growth rate of different organs is a power function of body size [7]. Allometric growth refers to the quantitative relationships between individual organs that grow at different rates [8, 9]. This concept has been broadly used to study size, shape, and function of plant organs, and to estimate multiple metabolic parameters [4, 10]. The total annual growth of a plant results from the increase in the size of different organs, making allometric relationships valid for different parts of the plant and throughout its life cycle [11, 12]. Allometric growth relies on the unequal allocation of biomass and is intrinsic to a species, being mostly determined by genetics, yet, it is also influenced by specific phenotypic traits [13]. The phenotypic variation between plants of the same species is not caused only by differences in environmental resources, plant size also contributes to this diversity by changing the allometric trajectory of individuals [4]. Therefore, allometric relationships can be used to predict plant growth and ecosystem functions and reflect plant plasticity in response to environmental changes [14].

*Fritillaria przewalskii* Maxim (Liliaceae). is an endangered perennial plant used in traditional Chinese medicine for the antibacterial and antitussive effects of its bulbs [15]. Rounder bulbs are preferred and sell at higher prices. Artificial cultivation is currently unavailable for this species, and overharvesting and the destruction of natural habitats are depleting its wild populations. They inhabit the harsh alpine meadows and grasslands located west of the Qinghai-Tibet Plateauat 3000 to 5000 m above the sea level [16]. The plants can be found in the Gannan Tibetan Autonomous Prefecture, and in the Counties of Zhangxian, Dingxi, Minxian and Weiyuan in Gansu province of northwest China. *F*. *przewalskii* plants are dormant most of the year; all growth happens over a 60 to 70 day period between the end of spring and beginning of summer. During 5 years of exclusive vegetative growth, plants are comprised of a fibrous root, a single bulbous geophyte, and produce only one leaf per year [17]. Bulbs are vital carbohydrate reserves that allow plants to survive dormant periods and buffer the impact of adverse environmental conditions [18]. They are particularly important for species that grow in harsh or unstable habitats, like *F*. *przewalskii*. Leaves have essential roles in plant physiology and long-term adaptation to environmental changes [19]. Leaf and bulb morphology and biomass accumulation are fundamental plant functional traits. They have been widely used to predict plant growth strategies and responses to the environment [20]. Plant growth follows a trade-off relationship between different organs [21], which implies that natural selection prioritizes some capabilities at the expense of others [4]. Therefore, researching leaf and bulb functional traits, growth relationships, and biomass allocation is fundamental for predicting ecosystem-level functions and processes.

Altitude is an easily measurable index parameter for various environmental factors, including air temperature, radiation, and soil nutrients. Increasing altitude associated with a decrease in atmospheric pressure and temperature, which directly affects photosynthesis [22, 23]. Altitudinal gradients can be used as model templates for large-scale studies that analyze the adaptive features of terrestrial plants under the influence of global climate change [23, 24]. Biomass peaks in the cold and wet environments associated with higher altitudes [25]. Altitude might influence the allometric relationships between leaf and bulb traits and biomass allocation, as these exhibit considerable plasticity in response to various environmental demands [26]. Awareness of local-scale variation in many leaf traits for individual species, as well as the relationships among these traits and their dependence on altitude, might be essential for extrapolating ecophysiological processes from the leaf to the ecosystem level [27].

The increasing demand for *F*. *przewalskii* and minimal yearly growth have rendered its wild resources nearly extinct. Therefore, it is fundamental to domesticate wild plants under artificial conditions and develop large-scale standardized cultivation procedures to meet the growing market demand. Yet, the knowledge of how *F*. *przewalskii* plants react to growth under different conditions is scarce. Namely, it is unknown how biomass allocation and leaf and bulb growth respond to changes in altitude. In this study, we analyzed the morphology and biomass allocation of leaves and bulbs of *F*. *przewalskii* growing at three altitudes (2400, 2700, 3000 m) in the Zhaishang Mountain in China. We determined the allometric relationships between leaf and bulb traits and biomass allocation. Finally, we asked if altitude changed the relationship between these traits or their allometric trajectories. With these analyses, we uncovered the allometric growth strategies that occur in response to changes in the environment and inferred how plasticity is used to adapt to different altitudes. By identifying the conditions in which plants achieve a better performance, we provide an essential theoretical basis for developing artificial cultivation techniques for *F*. *przewalskii*.

## Materials and methods

### Area of research

The Zhaishang Mountain is located in the Gansu province of China (34°32'-34°34'N, 104°15'E—-104°15'E), and the soil is classified as “black soil”. Its altitude ranges from 2200 to 3200 m. The vegetation varies with elevation: below 2700 m, most of the area is cropland; from 2700 to 3000 m, shrubs dominate the vegetation; and above this elevation, grassland are widespread and universal.

### Study design and sampling

Currently, artificial cultivation of *F*. *przewalskii* is not possible, and it is a level 3 protected plant. Sampling for experimental research was allowed according to the article 12 of the regulations on the protection of wild herb resources management. The necessary permits and approvals for performing this work were obtained by the natural science foundation of China, and also allowed by the Chinese ministry of science and technology.

In 2013, wild seeds of *F*. *przewalskii* were gathered yearly at autumn when they were ripe. Then they were sowed in the Zhaishang Mountain at an altitude gradient at three points: low, 2400±20; middle, 2700±20; and high, 3000±20 m. All the plants were grown in natural conditions, with consistent field management conditions, and no weeding and or application of fertilizers. Five years after sowing, 10 plants with consistent growth were harvested and used to study biomass allocation and leaf traits at each altitude at the end of the growing season (28 June 2018). At the same time, 50 plants with difference vegetative sizes were collected at each altitude and used to study allometric relationships between leaf and bulb traits. All plants were separated into roots, bulbs, and leaves. The leaf traits of length, thickness, and width, and the bulb horizontal and vertical dimensions were measured with rulers. Leaf length/width ratio and bulb horizonta/vertica were calculated from those measurements. Leaf area was measured with a scanner (Cano Scan LIDE 110, Japan). After measurement of traits, bulb and leaf samples were place in a drying oven for 48 h at 75°C, and their dry mass measured with an electronic balance. The total biomass was calculated as the sum of bulb and leaf biomass. Biomass allocation was calculated as the ratio between bulb or leaf biomass and the total value. Specific leaf area (SLA) and leaf area ratio (LAR) were calculated as SLA = leaf area/leaf dry weight and LAR = leaf area/total dry weight.

### Data analysis

The variation of leaf and bulb traits, biomass, and biomass allocation with altitude were tested for significance with one-way ANOVA (The LSD was used to test the difference) in SPSS 19.0. All values presented are means±SE. A reduced major axis (RMA) was performed using the SMART package (http://www.bio.mq.edu.au/ecology/SMART) to examine allometric relationships [28]. First, we used a scaling approach, with Y = aX^b^ to examine the allometric relationships of different traits. After log-transformation, the power function could be expressed using a linear regression equation, where a is the regression intercept and b is the regression slope [29, 30]. b = 1, indicates isometric growth, and b≠1 indicates allometric growth [31, 32]. b can be tested at different slope aspects by heterogeneity tests [33]. If the slope b is significantly different, it indicates the allometric growth trajectory changed. If there is no heterogeneity, a common slope can be presented in the equation. It can also be further analyzed whether there is variation in the Y-axis direction at the common slope. If there is a significant increase in the intercept a, it indicates that trajectory is changed and Y has a higher value at a given X value.

## Results

### Functional traits of leaves and bulbs grown at different altitudes

**Fig 1:**
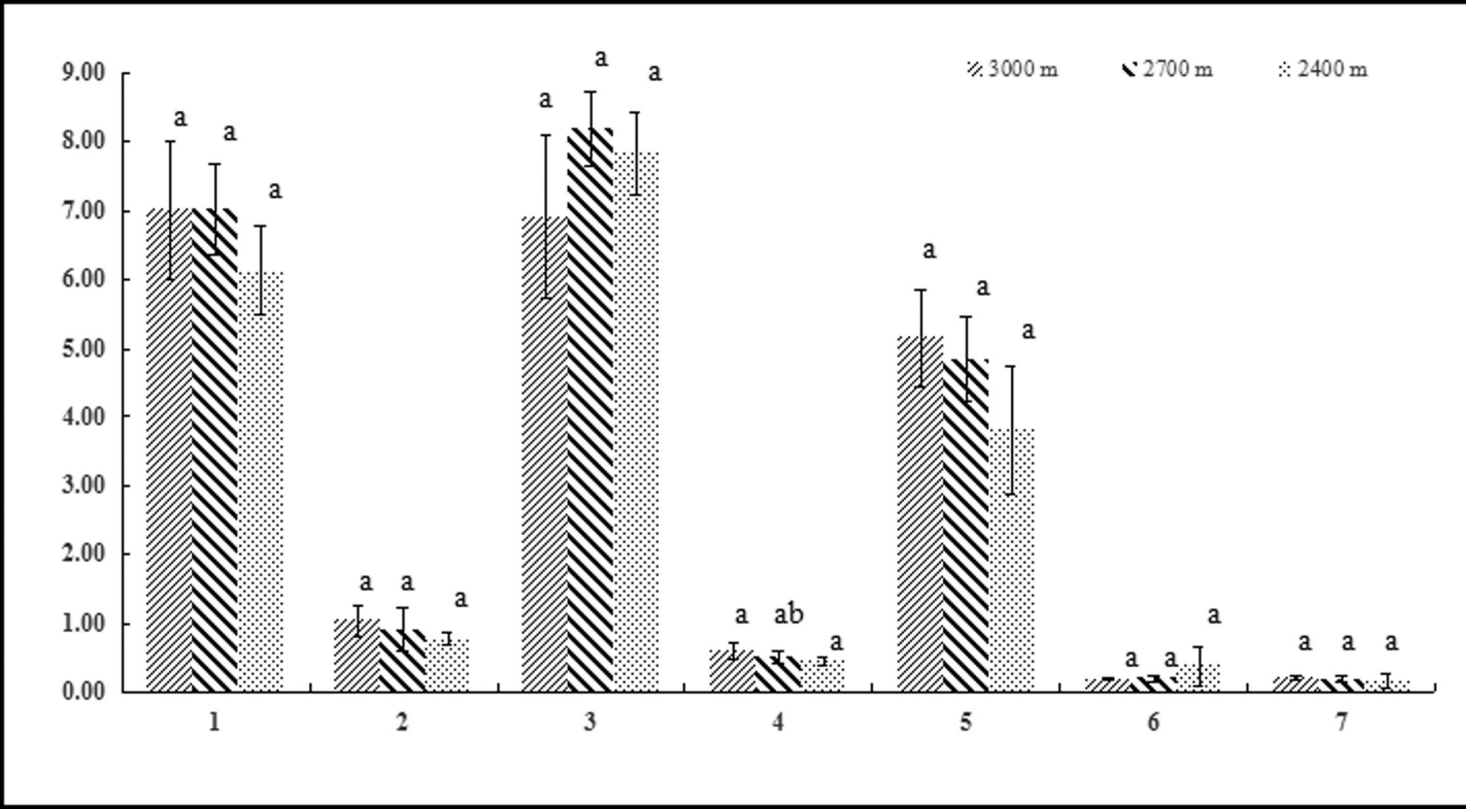
Leaf morphological traits of *F*. *przewalskii* at different altitudes, 1: Leaf length (cm), 2: Leaf width (cm), 3: Leaf length:width ratio, 4: Leaf thickness (mm), 5: Leaf area (cm^2^), 6: SLA (cm^2^·g^-1^), 7: LAR(cm^2^·g^-1^). Different small letters indicate a significant difference (P<0.05) at different altitudes.

**Fig 2:**
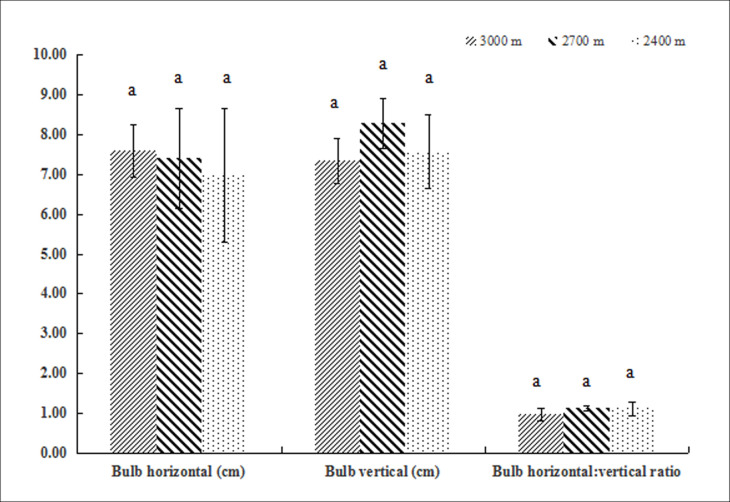
Bulb morphological traits of *F*. *przewalskii* at different altitudes, 1: Bulb horizontal (cm), 2: Bulb vertical (cm), 3: Bulb horizontal:vertical ratio. Different small letters indicate a significant difference (P<0.05) at different altitudes.

Leaf thickness correlated significantly and positively with altitude (P<0.05). None of the other leaf or bulb traits analyzed were affected by altitude (Figs 1 and 2).

### Biomass and allocation in leaves and bulbs grown at different altitudes

**Fig 3:**
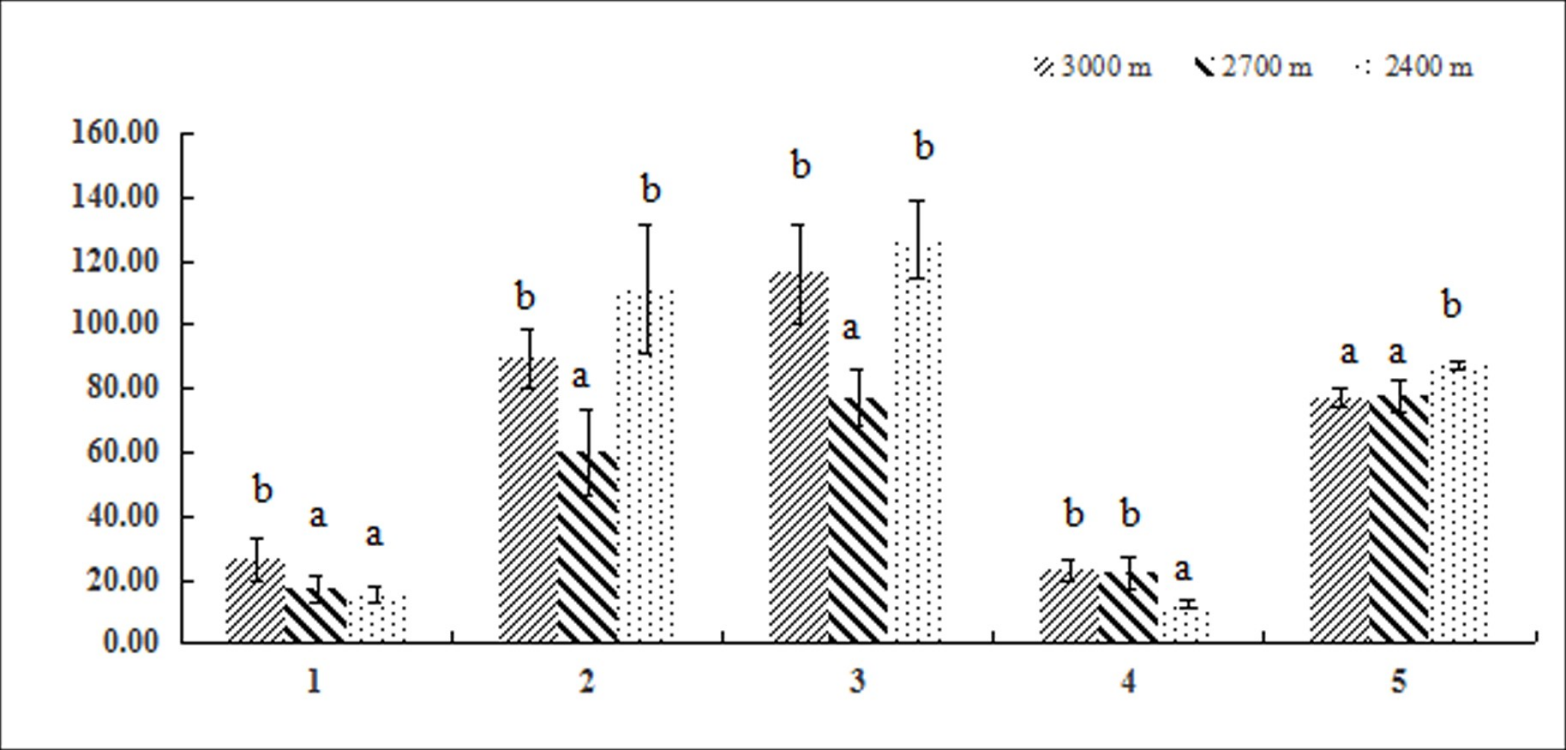
Biomass and biomass allocation in leaf and bulb of *F*. *przewalskii* at different altitudes, 1: Leaf biomass (mg), 2: Bulb biomass (mg), 3: Total biomass (mg), 4: Leaf biomass allocation (%), 5: Bulb biomass allocation (%). Different small letters indicate a significant difference (P<0.05) at different altitudes.

Leaf biomass was significantly higher at 3000 m than at 2700 or 2400 m. Bulb and total biomass were significantly lower at medium altitude (2700 m) than at the others, with the highest value occurring at 2400 m. Furthermore, the leaf:bulb ratio (0.14) and leaf allocation were significantly lower at 2400 m than at both high (0.3) and medium (0.29) altitude. Bulb biomass allocation also correlated negatively with altitude. Nevertheless, most of the biomass was allocated to bulbs at all tested altitudes (> 75% for all), confirming their essential role in carbohydrate storage. Hence, altitude increased leaf biomass but decreased it in bulb and total values (Fig 3).

### Relationship between leaf area and other functional traits

The correlation between leaf area and the length:width ratio was the highest at 3000 m. The effect was the opposite for thickness, which had the best correlation with the leaf area at the lowest altitude. Length, width, leaf biomass, and total biomass all had high correlations with the leaf area at all altitudes tested (R^2^, P<0.001). The correlation was positive with leaf length, thickness, width, and biomass, and total biomass. Conversely, the leaf area was negatively correlated with the length:width ratio.

The SMA slopes of the correlation between leaf area and length, thickness, width, and length:width were statistically different from 1.00 at all altitudes. This result indicated that these traits are allometrically related and that they are responsible for leaf area increase. There were no statistically significant differences within each correlation at different altitudes. The common slope was 2.061 for length, 4.043 for thickness, and -2.472 for the length:width ratio. The intercepts of leaf area with length and length:width on the y-axis were all not statistically different, while the leaf area with thickness was significantly different at different altitudes. For leaf area and leaf biomass and total biomass, the SMA slopes were not different from 1.00 at the three altitudes, indicating isometric growth. The SMA slopes for leaf area correlated with thickness were significantly higher for 2400 m than for 2700 or 3000 m. For the relation between leaf area and leaf biomass, the SMA slope was significantly greater at 3000 m than at 2400 or 2700 m. The SMA slopes for leaf area correlated with total biomass were not significantly different at different altitudes. The common slope was 0.954 on the y-axis, and the intercept was higher at 3000 m than at 2400 and 2700 (Table 1).

**Table 1:** Allometric relationship between leaf area (y) and other leaf functional traits (x) in *F*. *przewalskii* grown at different altitudes.

traits	altitude (m)	N	R^2^	SMA Slope	P Compare to 1	Common slope	Intercept	95% confidence interval
**length**	1	50	0.783**	2.274a	0.000	2.061	-1.011a	1.987–2.603
	2	50	0.818**	2.134a	0.000		-1.057a	1.887–2.415
	3	50	0.909**	1.943a	0.000		-1.053a	1.780–2.120
**thick**	1	50	0.156*	3.777a	0.000	4.043	1.359c	2.902–4.916
	2	50	0.329**	4.130a	0.000		1.535b	3.264–5.227
	3	50	0.374**	4.167a	0.000		1.772a	3.319–5.233
**width**	1	50	0.896**	1.493b	0.000	-	-	1.360–1.640
	2	50	0.846**	1.651b	0.000			1.474–1.850
	3	50	0.858**	1.978a	0.000			1.773–2.206
**length:width ratio**	1	50	0.263**	-2.095a	0.000	-2.472	2.500a	-2.681–1.637
	2	50	0.102*	-2.394a	0.000		2.486a	-3.141–1.824
	3	50	0.005	3.207a	0.000		2.365a	2.411–4.267
**leaf biomass**	1	50	0.965**	1.0554a	0.052	-	-	0.9995–1.1144
	2	50	0.956**	0.9564b	0.145	-	-	0.9002–1.0161
	3	50	0.450**	0.9008b	0.333	-	-	0.7276–1.1153
**total biomass**	1	50	0.914**	0.9810a	0.652	0.954	-1.268a	0.9009–1.0681
	2	50	0.771**	0.9065a	0.161		-1.410b	0.7893–1.0411
	3	50	0.556**	0.9104a	0.333		-1.421b	0.7512–1.1033

Note: 1, 2, 3 are altitudes of 3000 m, 2700 m, and 2400m, respectively.

* and ** indicate significant differences between altitudes with P<0.05 and P<0.001, respectively.

Different small letters indicate a significant difference (P<0.05) in the SMA slope or intercept between altitudes.

### Relationship between leaf biomass and other functional traits

The correlation between leaf biomass and length:width decreased with the decrease in altitude, but was overall low. Regarding leaf thickness, the correlation with biomass was low but was the highest at 2700 m and very low for 2400 m. Leaf biomass correlated very positively with length and width, particularly at the highest altitude (R^2^, P<0.001) (Table 2).

**Table 2:** Allometric relationships between leaf biomass (y) and other leaf functional traits (x) in *F*. *przewalskii* grown at different altitudes.

traits	Altitude(m)	N	R^2^	Slope	P Compare to 1	Common slope	Intercept	95% confidence interval
**length**	1	50	0.769**	2.155a	0.000	2.187	-0.377a	1.875–2.476
	2	50	0.782**	2.232a	0.000		-0.441a	1.950–2.554
	3	50	0.549**	2.157a	0.000		-0.454a	1.777–2.618
**thickness**	1	50	0.128*	3.579a	0.000	4.155	2.106c	2.738–4.677
	2	50	0.325**	4.318a	0.000		2.268b	3.410–5.469
	3	50	0.050	4.626a	0.000		2.490a	3.499–6.116
**width**	1	50	0.863**	1.415b	0.000	-	-	1.271–1.575
	2	50	0.802**	1.727a	0.000		-	1.518–1.964
	3	50	0.328**	2.195a	0.000		-	1.734–2.779
**length:width ratio**	1	50	0.245**	-1.985b	0.000	-	-	-2.547–1.547
	2	50	0.094*	-2.503ab	0.000		-	-3.288–1.905
	3	50	0.086*	3.560a	0.000		-	2.707–4.682
**total biomass**	1	50	0.918**	0.9295a	0.083	0.949	-0.538a	0.8554–1.0099
	2	50	0.830**	0.9478a	0.372		-0.703b	0.8413–1.0679
	3	50	0.767**	1.0106a	0.880		-0.740b	0.8790–1.1619

Note: 1, 2, 3 are altitudes of 3000 m, 2700 m, and 2400m, respectively.

* and ** indicate significant differences between altitudes with P<0.05 and P<0.001, respectively.

Different small letters indicate a significant difference (P<0.05) in the SMA slope or intercept between altitudes.

The SMA slopes for leaf biomass correlated with length, thickness, width, and length:width ratio was significantly different from 1 at different altitudes. This result indicates that they represent allometric growth, with these traits being responsible for the accumulation of leaf biomass. The SMA slopes for the correlation between leaf and total biomass were not statistically different from 1.00 at different altitudes, indicating isometric growth. The SMA slopes for leaf biomass with length, thickness, and total biomass were all not significantly changed at different altitudes. The common slopes were 2.187, 4.155, and 0.949, respectively. The intercept of leaf biomass with length on the y-axis were all not significantly different. Thickness was significantly different at different altitudes, with total biomass at 3000 m significantly higher than at 2700 and 2400 m. The SMA slopes for leaf biomass correlated width and length:width significantly more at 3000 m significantly than at 2400 (Table 2).

### Relationship between bulb biomass and other functional traits

The correlation between bulb biomass and the horizontal:vertical ratio of bulbs decreased with a decrease in altitude. Their correlation was negative. Bulb biomass correlated very positively with horizontal and vertical dimensions at different altitudes (R^2^, P<0.001). The SMA slopes for bulb biomass correlated with horizontal and vertical dimensions, and the horizontal:vertical ratio was statistically different from 1.00 at different altitudes, indicating allometric growth. The difference between the SMA slopes for bulb biomass correlated with horizontal dimensions were significantly higher at 2700 m. Altitude did not significantly change the values of the correlation with the vertical dimensions. The common slope was 3.084. The intercept at 3000 m (-0.745) was significantly higher than at 2400 (-0.872) and 2700 m (-0.849). Altitude affected the correlation between bulb biomass and the horizontal:vertical ratio. The highest value occurred for the middle altitude (10.342), and the lowest for the 3000 m (4.311) (Table 3). All these differences were statistically significant.

**Table 3:** Allometric relationship between bulb biomass (y) and other bulb functional traits (x) in *F*. *przewalskii* grown different altitudes.

traits	Altitude(m)	N	R^2^	SMA Slope	P Compare to 1	Common slope	Intercept	95% confidence interval
**horizontal**	1	50	0.919**	2.412b	0.000	-	-	2.221–2.620
	2	50	0.945**	3.426a	0.000		-	3.202–3.666
	3	50	0.390**	2.976ab	0.000		-	2.377–3.726
**vertical**	1	50	0.821**	2.968a	0.000	3.083	-0.745b	2.928–3.741
	2	50	0.933**	3.309a	0.000		-0.849a	2.753–3.200
	3	50	0.430**	3.397a	0.000		-0.872a	2.734–4.222
**horizontal:vertical ratio**	1	50	0.285**	-4.311b	0.000	-	-	-5.497–3.381
	2	50	0.193**	10.342a	0.000		-	7.991–13.383
	3	50	0.020	-8.383c	0.000		-	-11.131–6.313

Note: 1, 2, 3 are altitudes of 3000 m, 2700 m, and 2400m, respectively.

* and ** indicate significant differences between altitudes with P<0.05 and P<0.001, respectively.

Different small letters indicate a significant difference (P<0.05) in the SMA slope or intercept between altitudes.

### Relationship between bulb and total biomass and leaf functional traits

Bulb biomass was highly positively correlated with leaf area, leaf biomass, and total biomass at different altitudes (R^2^, P<0.001). The SMA slopes for leaf area and leaf biomass were not significantly different to 1 at different altitudes, indicating isometric growth. At the common slope, the intercept of bulb biomass with leaf area and leaf biomass on the y-axis were all significantly lowers at 3000 m than at the low and middle altitudes. The SMA slopes for bulb biomass correlated with total biomass at high and middle altitudes were significantly greater than 1, indicating allometric growth. At 2400 m, there was no difference to 1.00, indicating isometric growth. This suggests that the accumulation of total biomass triggered the increase in bulb biomass at high and middle altitudes. While altitude did not affect the SMA slopes, the common slope was 1.040 and intercept at 3000 m was significantly inferior than at 2700 m (Table 4).

**Table 4:** Allometric relationship between bulb biomass (y) and total biomass and leaf functional traits (x) in *F*. *przewalskii* grown different altitudes.

traits	Altitude(m)	N	R^2^	SMA Slope	P Compare to 1	Common slope	Intercept	95% confidence interval
**leaf area**	1	50	0.870**	1.061a	0.259	1.095	1.189b	0.956–1.178
	2	50	0.697**	1.146a	0.092		1.381a	0.977–1.344
	3	50	0.578**	1.140a	0.167		1.389a	0.945–1.375
**leaf biomass**	1	50	0.867**	1.120a	0.036	1.099	0.394b	1.008–1.245
	2	50	0.757**	1.096a	0.204		0.612a	0.950–1.264
	3	50	0.504**	1.027a	0.794		0.649a	0.838–1.258
**total biomass**	1	50	0.993**	1.041a	0.002	1.040	-0.191a	1.016–1.067
	2	50	0.991**	1.039a	0.007		-0.155b	1.011–1.067
	3	50	0.917**	1.038a	0.376		-0.160ab	0.955–1.129

Note: 1, 2, 3 are altitudes of 3000 m, 2700 m, and 2400m, respectively.

* and ** indicate significant differences between altitudes with P<0.05 and P<0.001, respectively.

Different small letters indicate a significant difference (P<0.05) in the SMA slope or intercept between altitudes.

### Relationship between bulb horizontal and vertical dimensions

Bulb horizontal and vertical dimensions correlated very positively at different altitudes (R^2^, P<0.001). The SMA slopes at 3000 and 2400 m were significantly higher to 1, indicating allometric growth. At the middle altitude, the value was significantly inferior to 1. The SMA slope significantly changed at different altitudes, with the biggest value occurring at 3000 m, and the smallest at 2700 (Table 5).

**Table 5:** Allometric relationship between bulb horizontal and vertical measurements in *F*. *przewalskii* grown at different altitudes.

bulb	Altitude(m)	N	R^2^	SMA Slope	P Compare to 1	Common slope	Intercept	95% confidence interval
**horizonta-vertica**	1	50	0.699**	1.3718a	0.000			1.1707–1.6074
	2	50	0.927**	0.8665c	0.001			0.8013–0.9369
	3	50	0.878**	1.1415b	0.011			1.0315–1.2632

Note: 1, 2, 3 are altitudes of 3000 m, 2700 m, and 2400m, respectively.

* and ** indicate significant differences between altitudes with P<0.05 and P<0.001, respectively.

Different small letters indicate a significant difference (P<0.05) in the SMA slope or intercept between altitudes.

## Discussion

Altitude is an important ecological factor that influences critical environmental parameters, including temperature and water availability. Hence, it has a major effect on modulating how plant populations differentiate [34]. *F*. *przewalskii* is a Liliaceae that grows in harsh alpine conditions at high altitudes, in the Gansu region of China. In this study, we investigated how growing *F*. *przewalskii* along an altitude gradient affects the development of above-ground leaves and underground bulbs. This type of analysis can help us better understand how plants actively adapt to variations in their environment [20, 35]. Our results indicated that altitude does not affect multiple morphological characteristics of leaves, including length, width, length:width ratio, leaf area, specific leaf area, and leaf area ratio. The same is true for the horizontal and vertical axes of bulbs. The only trait impacted was leaf thickness, which significantly increased with altitude (3000 meters). It is known that plants increase leaf thickness to adapt to the low temperatures inherent of high altitudes [36]. An increase in thickness reduces plant transpiration, improves water storage capacity, and enhances photosynthetic efficiency.

The allocation of resources to different organs during plant ontogeny is fundamental for correct growth and response to the environment [4]. Most studies look at biomass because it reflects the patterns of multiple pathways [37]. Change in the proportion of biomass allocated among organs indicates an adjustment to the plant growth strategy. Therefore, the analysis of allocation patterns is useful for investigating plants’ priorities under varying environmental conditions. We found that the allocation of biomass to leaves increased significantly with altitude, while it decreased for bulbs. This response suggests that the variation in altitude alters the resource allocation strategy of *F*. *przewalskii* plants. The intensity of light radiation increases with altitude, and the conditions become more adverse for plants. To prevent excessive water loss and efficiently fight pests, plants prioritize the growth and thickening of leaves. This change increases the rate of absorption and assimilation of resources and of photosynthate accumulation. More resources become available for plant defense [38]. Yet, the pattern of biomass allocation was unchanged among the tested altitudes, with bulbs always supplanting leaves. The allocation of most biomass to underground organs is a common strategy in perineal plants [39]. Kleijn et al. proposed that the existence of specialized storage organs, including bulbs, contributes to the response to adverse conditions [18].

The theory rooted in metabolic ecology suggests that allometric growth models can explain the trade-off between different plant organs [40–42]. Allometric growth refers to the difference in the relative growth rates of two characters of an organism. It is not only determined by species genetics but also influenced by the surrounding environmental factors [4, 43, 44]. Hence, it reflects both the structural and functional characteristics of organisms, and their relationship with each other, and the ecological strategies adopted by different components when facing variation in resources [45]. The different growth rates of the various plant organs lead to differences in phenotypes, namely size, among individuals of the same species. This variation is not only the result of the response to different growth conditions and resources but depends on the total biomass of the plants [4, 46]. The variation of the plant’s allometric growth trajectory is the manifestation of the adaptation of plants to the resources. Our results show that the allometric growth trajectory of the leaf area in relation with length and the length:width ratio did not change significantly with altitude. This indicated that their growth was caused by plant size as the result of phenotypic plasticity. However, altitude changed the relation between leaf area and thickness, width, biomass, and total biomass. Specifically, while the leaf area is constant, the leaf thickness increased with the altitude, and leaf and total plant biomass decreased. The change in allometric relationships between leaf traits are both a cause and a consequence of variations in resource availability and environmental changes [47, 48].

Our analyzes also showed that altitude does not affect the allometric growth trajectory between leaf biomass and length, indicating that variation in the growth rate at different altitudes is likely the result of the phenotypic plasticity driven by the total biomass of the plants. Conversely, the variation of altitude drove the change in the correlation with leaf thickness, width, aspect ratio, and total biomass. Altitude also impactd the allometric growth trajectory between the bulb biomass and horizontal dimensions, vertical dimensions, and the horizontal:vertical ratio. The correlation with the horizontal dimensions was more significant at the highest altitude tested than at the others. The effect was the opposite for the vertical bulb dimensions. Consequently, the horizontal:vertical ratio was lowest at the medium altitude. Additionally, altitude changed the allometric growth trajectory between bulb biomass and leaf area, leaf biomass, and total biomass. In particular, the correlation with leaf area and leaf biomass was higher at high altitude, similarly to what happened for total biomass. The allometric correlation between the horizontal and vertical dimensions of the bulb was largest at high altitude and lowest at the midpoint.

In summary, *F*. *przewalskii* adapts to growing at high altitude by changing morphological and growth characteristics. As seen for other plants and ecosystems, those changes are not only the result of adaption to heterogeneous environments but allow plant organs to improve their performance through manipulating the phenotypic plasticity of their structures. Through these changes, they increase the utilization of light and nutrient resources at high altitudes, providing an effective protection mechanism for themselves simultaneously [49].

## Conclusion

In this study, we found that the altitude has a considerable effect on the growth and morphological characteristics of *F*. *przewalskii* plants. *F*. *przewalskii* adapts to high altitude environments by increasing the width and thickness of its leaves. Furthermore, the total biomass of the plants decreases with altitude, and the bulb promotes vertical growth. The altitude influenced the allometric growth trajectories of specific leaf and bulb traits: higher altitudes led to thicker and broader leaves and changed the shape of the bulbs from more circular, which is ideal (at 2700 m), to more elongated (at 3000 m), those variations had remarkable ecological significance. Bulb biomass is the largest at 2700 m of altitude for which their vertical and longitudinal ratio is unaffected, so 2700 m is the most suitable altitude for its artificial cultivation. Our findings suggest that *F*. *przewalskii* has a great potential of growth and morphological plasticity.

## Supporting information

S1 Data (RAR)

## References

[1] Müller I, Schmid B, Weiner J. The effect of nutrient availability on biomass allocation patterns in 27 species of herbaceous plants. Perspectives in Plant Ecology, Evolution and Systematics. 2000; 3: 115–127.

[2] Wang J S, Zhang C Y, Fan X H. Biomass Allocation Patterns and Allometric Models of *Abies nephrolepis Maxim*. Acta Ecologica Sinica. 2011; 31: 3918–3927.

[3] White J. The allometric interpretation of the self-thinning rule. Journal of theoretical Biology. 1981; 89: 475–500.

[4] Weiner J. Allocation, plasticity and allometry in plants. Perspectives in Plant Ecology Evolution and Systematics. 2004; 6: 207–215.

[5] Caswell H, Salguero-Gómez R. Age, stage and senescence in plants. Journal of Ecology. 2013; 101: 585–595. 10.1111/1365-2745.12088 23741075 PMC3664411

[6] Fan X, Lin Z, Ding X, Zhu J. Sexual size dimorphism and female individual fecundity of *Silurus asotus* and *Clarias fuscus*. Acta Ecologica Sinica. 2014; 34: 555–563.

[7] Huxley J. Constant differential growth-ratios and their significance. Nature.1924; 114: 895–896.

[8] Dong D, Lin T X, Tang J Y. Biomass Allocation Patterns and Allometric Models of *Tilia amurensis*. Journal of Beijing Forestry University. 2014; 36: 54–63.

[9] Enquist B J, West G B, Charnov E L, Brown J H. Allometric scaling of production and life- history variation in vascular plants. Nature. 1999; 65: 3529–3538.

[10] Voje K L, Hansen T F, Egset C K, Bolstad G H, Pelabon C. Allometric constraints and the evolution of allometry. Evolution. 2014; 68: 866–885. 10.1111/evo.12312 24219593

[11] Niklas K J. Growth and metobolism Plant allometry: The scaling of plant form and process. 1st ed. (pp. 1–60). 1994; London, IL, USA: The University of Chicago Press.

[12] Niklas K J. Plant allometry: Is there a grand unifying theory? Biological Reviews. 2004; 79: 871–889. 10.1017/s1464793104006499 15682874

[13] Paul K I, Roxburgh S H, Chave J, England J R, Zerihun A, Specht Paul K I, Roxburgh S H, Chave J, England J R, Zerihun A, Specht A. Testing the generality of aboveground biomass allometry across plant functional types at the continent scale. Global Change Biology. 2016; 22: 2106–2124. 10.1111/gcb.13201 26683241

[14] Chave J, Réjou-Méchain M, Búrquez A, Chidumayo E, Colgan M S, Duque A. Improved allometric models to estimate the aboveground biomass of tropical trees. Global Change Biology Bioenergy. 2014; 20: 3177–3190. 10.1111/gcb.12629 24817483

[15] Li H J, Jiang Y, Li P. Characterizing distribution of steroidal alkaloids in *Fritillaria* spp. and related compound formulas by liquid chromatography-mass spectrometry combined with hierarchial cluster analysissJ. Journal of Chromatography A. 2009; 1216:2142–2149. 10.1016/j.chroma.2008.03.093 18462741

[16] Guo F X, Chan Y L, Lin Y H. Studies on grain filling characteristics of *Fritillaria przewalskii*. Acta Prataculturae Sinica. 2010; 19: 97–102.

[17] Chang Y L, Chen Y, Guo F X, Lin Y H, Li T. Study on water absorption and germination characteristics in seeds of *Fritillaria przewalskii Maxim*. Acta Prataculturae Sinica. 2010; 19: 41–46.

[18] Kleijn D, Treier U A, Müller-Schärer H. The importance of nitrogen and carbohydrate storage for plant growth of the alpine herb Veratrum album. New Phytologist. 2005; 166: 565–575. 10.1111/j.1469-8137.2005.01321.x 15819918

[19] Misgana Z H. Modeling Leaf Area Estimation for Arabica Coffee (*Coffea arabica* L.) Grown at Different Altitudes of Mana District, Jimma Zone. American Journal of Plant Sciences. 2018; 9: 1292–1307.

[20] Guo Z W, Lin H, Chen S L, Yang Q P. Altitudinal patterns of leaf traits and leaf allometry in Bamboo *Pleioblastus amarus*. Frontiers in plant science. 2018; 9: 1110–1117. 10.3389/fpls.2018.01110 30108603 PMC6080356

[21] Liang K L, Zhang H R, Zhang L J. Response of phenotypic plasticity and plant resource allocation of Amorpha fruiticosa to alpine habitat. Pratacultural Science. 2012; 29: 440–446.

[22] Hunter J T. Changes in Allometric Attributes and Biomass of Forests and Woodlands across an Altitudinal and Rainfall Gradient: What Are the Implications of Increasing Seasonality due to Anthropogenic Climate Change? International Journal of Ecology. 2015; 1–10.

[23] Li M H, Jiang Y, Wang A, Li X, Zhu W, Yan C F. Active summer carbon storage for winter persistence in trees at the cold alpine treeline. Tree Physiology. 2018; 38: 1345–1355. 10.1093/treephys/tpy020 29538773

[24] Wang A, Wang X, Tognetti R, Lei J P, Pan H L, Liu X L, et al Elevation alters carbon sinks, nutrient concentrations and stoichiometry in *Quercus aquifolioides* in southwestern China. Science Total Environment. 2018; 622: 1463–1475. 10.1016/j.scitotenv.2017.12.070 29890611

[25] Culmsee H, Leuschner C, Moser G, and Pitopang R. Forest aboveground biomass along an elevational transect in Sulawesi, Indonesia, and the role of Fagaceae in tropical montane rain Forests. Journal of Biogeography. 2010; 37: 960–974.

[26] Valladares F, Gianoli E, Gómez J M. Ecological limits to plant phenotypic plasticity. New Phytologist. 2007; 176: 749–763. 10.1111/j.1469-8137.2007.02275.x 17997761

[27] Navas M L, Roumet C, Bellmann A, Laurent G, Garnier E. Suites of plant traits in species from different stages of a Mediterranean secondary succession. Plant Biology. 2010; 12: 183–196. 10.1111/j.1438-8677.2009.00208.x 20653901

[28] R Development Core Team. R: A Language and Environment for Statistical Computing. 2013; Vienna: R Foundation for statistical computing.

[29] Kerkhoff A J, Fagan W F, Elser J J, Enquist B. Phylogenetic and growth form variation in the scaling of nitrogen and phosphorus in the seed plants. The American Natutalist. 2006; 168: 103–122. 10.1086/507879 17004214

[30] Warton D I, Wright I J, Falster D S, Westoby M. Bivariate line-fitting methods for allometry. Biological Reviews. 2006; 81: 259–291. 10.1017/S1464793106007007 16573844

[31] Niklas K J, Enquist BJ. On the vegetative biomass partitioning of seed plant leaves, stems, and roots. American Naturalist. 2002; 59: 482–497. 10.1086/339459 18707431

[32] Niklas K J. 2005 Modelling below- and above-ground biomass for non-woody and woody plants. *Annals of Botany* 95: 315–321. 10.1093/aob/mci028 15546927 PMC4246831

[33] Warton D I, Weber N C. Common slope tests for bivariate errors-in-variables models. Biometrical Journal. 2002; 44: 161–174.

[34] Hong L P, Maihe L, Xiao H C. Responses of growth and ecophsiology of plants to altitude. Ecology and Environmental Sciences. 2009; 18: 722–730.

[35] Craine J M, Lee W G, Bond W J, Williams R J, Johnson L C. Environmental constraints on a global relationship among leaf and root traits of grasses. Ecology. 2005; 86: 12–19.

[36] Wang J, Gao J, Wu Y, Sun J, Xu B, Shi F, Bisht N, Xu J, Wu N. Biomass allocation and trade-offs of *Pedicularis longiflora Rudolph*. at two slope aspects in an alpine meadow of the eastern Tibetan Plateau. Applied Ecology and Environmental Research. 2017; 15: 51–65.

[37] Niinemets U, Portsmuth A, Tobias M. Leaf size modifies support biomass distribution among stems, petioles and mid-ribs in temperate plants. New Phytologist. 2006; 171: 91–104. 10.1111/j.1469-8137.2006.01741.x 16771985

[38] Vogel S. Leaves in the lowest and highest winds: temperature, force and shape. New Phytologist. 2009; 183: 13–26. 10.1111/j.1469-8137.2009.02854.x 19413689

[39] Tilman D. Plant Strategies and the Dynamics and Structure of Plant Communities. Princeton 1988; USA: Princeton University Press: 37–52.

[40] Niklas K J, Enquist BJ. Invariant scaling relationships for interspecific plant biomass production rates and body size. Proceedings of the National Academy of Sciences of the United States of America. 2001; 98: 2922–2927. 10.1073/pnas.041590298 11226342 PMC30241

[41] Savage V M, Deeds E J, Fontana W. Sizing up allometric scaling theory. Plos Computational Biology. 2008; 4: 17. 10.1371/journal.pcbi.1000171 PMC2518954 18787686

[42] West G B, Brown J H, Enquist B J. The fourth dimension of life: Fractal geometry and allometry scaling of organisms. Science. 1999; 284: 1677–1679. 10.1126/science.284.5420.1677 10356399

[43] Huang Y X, Zhao X Y, Zhang H X, Japhet W, Zuo X A, Luo Y Y, et al Allometric effects of Agriophyllum squarrosum in response to soil nutrients, water, and population density in the Horqin sandy land of China. Journal of Plant Biology. 2009; 52: 210–219.

[44] Yan X H, He C L, Zhou B. Biomass Distribution and Allometric Analysis of *Bidens frondosa* Relative to Growth Stage. Journal of Ecology and Rural Environment. 2017; 33: 150–158.

[45] Reekie E G, Bazzaz F A. Reproductive effort in plants. 2. Does carbon reflect allocation of other resources? The American Naturalist. 1987; 129: 897–906.

[46] Li Y, Zhao C Z, Hou Z J, Ma X L, Zhang Q. Body size and stem-and leaf allometry of *Stellera chamaejasme* in degraded alpine grassland. Chinese Journal of Ecology. 2013; 32: 241–246.

[47] Forrester D I, Tachauer I H H, Annighoefer P, Barbeito I, Pretzsch H, Ruiz-Peinado R. Generalized biomass and leaf area allometric equations for European tree species incorporating stand structure, tree age and climate. Forest Ecology and Management. 2017; 396: 160–175.

[48] Zhang T, Zhao Y L, Jin H, Zhang J Y, Wang Y Z. Morphological variability and allometric relationships of the herb Panax notoginseng in Yunnan, China. Acta Ecologica Sinica. 2017; 37: 65–69.

[49] Brodribb T J, Feild T S, Sack L. Viewing leaf structure and evolution from a hydraulic perspective. Functional Plant Biology. 2010; 37: 488–498.

