# Correction: Allometric relationships between leaf and bulb traits of *Fritillaria przewalskii* Maxim. grown at different altitudes

**DOI:** 10.1371/journal.pone.0253352

**Published:** 2021-06-10

**Authors:** Ruili Ma, Shengrong Xu, Yuan Chen, Fengxia Guo, Rui Wu

There is an error in affiliation 1 for authors Ruili Ma and Shengrong Xu. The correct affiliation 1 is: Dingxi Academy of Agricultural Sciences, Dingxi, China.
