# Correction: Allometric relationships between leaf and bulb traits of *Fritillaria przewalskii* Maxim. grown at different altitudes

**DOI:** 10.1371/journal.pone.0264810

**Published:** 2022-02-25

**Authors:** The PLOS ONE Staff

There are multiple errors in the author affiliations in the original article and previous correction notice. The correct affiliations are as follows:

Ruili Ma^1,2,3^, Shengrong Xu^1,2^, Yuan Chen^2*^, Fengxia Guo^2*^, Rui Wu^2^

**1** Qinghai University Medical College, Qinghai Provincial Key Laboratory of Traditional Chinese Medicine Research for Glucolipid Metabolic Diseases, Xi’ning, China, **2** College of Agronomy, Gansu Provincial Key Laboratory of Aridland Crop Science, Gansu Key Laboratory of Crop Genetic & Germplasm Enhancement, Gansu Provincial Key Lab of Good Agricultural Production for Traditional Chinese Medicines, Gansu Provincial Engineering Research Centre for Medical Plant Cultivation and Breeding, College of Life Science and Technology, Gansu Agricultural University, Lanzhou, China, **3** Dingxi Academy of Agricultural Sciences, Dingxi, China.

The publisher apologizes for the error.
